# Neonatal Outcome from Triplet Interval Delayed Delivery: A Case Report

**DOI:** 10.1155/2013/451360

**Published:** 2013-12-09

**Authors:** Monika Lachowska, Dorota Paluszyńska, Tomasz Fuchs, Robert Woytoń, Mariusz Zimmer, Barbara Królak-Olejnik

**Affiliations:** ^1^Department of Neonatology, Medical University of Wrocław, ul. Borowska 213, 50-556 Wrocław, Poland; ^2^Department of Obstetrics and Gynecology, Medical University of Wrocław, ul. Borowska 213, 50-556 Wrocław, Poland

## Abstract

In the past decades, we have observed a large increase in the number of multifetal pregnancies, which is mainly associated with the introduction of assisted reproductive techniques. Even though neonatal intensive care of very premature infants has improved significantly, the risk of mortality and long-term morbidity is still much higher among these newborns. A longer interdelivery period may reduce perinatal mortality and morbidity. The authors report the case of a delayed interval delivery in trichorionic, triamniotic triplet pregnancy. After the labor of the first fetus in the 22nd week of gestation, a 75-day interval was achieved before the delayed delivery. To save the surviving fetuses, the umbilical cord was ligated at the cervical level immediately after the first delivery. The patient received antibiotics, tocolytics, and corticosteroids. A baby boy who weighed 1750 g and a girl who weighed 1700 g were successfully delivered by cesarean section in the 33rd week of pregnancy. The babies were discharged home at the age of 28 days. A follow-up examination 20 weeks later showed that their neurological development was normal and without any major problems. The maternal postpartum course was uneventful; the patient stayed in hospital taking care of the babies.

## 1. Introduction

In the past decades, a significant increase in multifetal pregnancies has been observed, which is predominantly associated with the introduction of assisted reproductive techniques. Multiple gestation brings in a risk of perinatal and postnatal complications both for the mother and her babies [1]. The main neonatal complications in these pregnancies result from prematurity. Despite, significant improvements in neonatal intensive care of very premature infants, these newborns still have a significantly higher risk of mortality and long-term morbidity. Perinatal mortality rates in developed countries range from 47 to 120 per 1,000 births for twins and from 93 to 203 per 1,000 births for triplets [2] and are strictly associated with the gestational age. A delay of 2 or more days in the premature delivery in newborns born before 30 weeks of gestation is associated with improved infant survival and higher infant birth weight [3].

Delayed interval delivery is reported when one or more fetuses are delivered vaginally and the remaining ones are retained in uterus. Since the first case of interval delivery described by Carson in 1880, a number of case reports have been published describing trials of postponing the delivery of remaining twins, triplets, and other multiples after the first premature delivery [2–18]. One of the most important factors which influence neonatal outcome is the duration of intrauterine stay of the retained sibling(s) after the delivery of the first one(s). The prolonged interdelivery period may reduce perinatal mortality and morbidity [4]. We report a case of extremely delayed interval delivery in triamniotic, trichorionic triplet pregnancy. After the labor of the first fetus in the 22nd week of gestation, the 75-day interval has been achieved before the delayed delivery.

## 2. Case Report

A 31-year-old primigravida was admitted to hospital at 21 weeks and 5 days of a triplet pregnancy because of clinical signs of threatened preterm labor. In the same day, she started to have premature contractions. The patient had become pregnant spontaneously without ovarian stimulation. The ultrasound examination which was administered routinely at the 21st week of gestation showed triamniotic, trichorionic pregnancy with the growth diagnosed as normal for the gestational fetal age. Soon after the admission to hospital, a rapid delivery of the presented fetus occurred, a 310 g male neonate was born alive; but died shortly after the birth due to immaturity. Immediately after the delivery of the first triplet, high ligation of the umbilical cord was performed to minimize the risk of intrauterine infection, and the uterine contractions ceased. The placenta of the first aborted fetus was lying on the posterior wall, while the placentas of the second and third triplets were located high on the anterior wall of the uterus. We did not observe any abnormalities of placentas and umbilical cords of the second and third triplets in the whole period of observation, and the placenta of the first aborted foetus did not show any signs of abruption. A delayed-interval delivery procedure was considered after any signs of chorioamnionitis were excluded. An informed consent was obtained from the patient regarding the risks of delaying further delivery. The patient was placed in the Trendelenburg position. Prophylactic intravenous tocolysis with *β*-sympathomimetics (fenoterol) was initiated, which after 24 hours was continued orally (fenoterol, nifedipine), and simultaneously antibiotics (cefuroxime 3 × 500 mg) were initiated in order to prevent infections. In addition, at the 24th week corticosteroids, were administered to induce fetal lung maturity (12 mg betamethasone intramuscularly every 12 h twice). The patient was continuously monitored through clinical assessment (blood pressure, heart rate, and temperature) and laboratory tests (complete blood cell count, C-reactive protein (CRP), haemostatic condition, and urine test). At weekly intervals cervical, and vaginal cultures were taken. Positive cervical cultures of *Enterococcus faecalis* were found at the 25th and 28th gestational weeks and treated successfully with intravenous ampicillin. The culture taken at the 31st week of gestation showed high-level aminoglycoside-resistant (HLAR) *Enterococcus faecalis*. During that period, the patient received only Chlorquinaldole and Metronidazole vaginally. Fetal monitoring consisted of biophysical assessment, including transabdominal ultrasound every 14 days and cardiotocography every 48 h starting at the 28th week, with no abnormal findings noted.

The pregnancy was terminated by caesarean section 75 days later (32 + 2 week) due to uterine contractions. Twin babies, a boy and a girl, were born with Apgar scores of 6 and 7 at 1 and 5 minutes, respectively, for both. The baby boy weighed 1750 g, which corresponded to the 40th centile for the gestational age, and the girl weighed 1700 g (30th centile). Due to persistent respiratory distress the babies were given continuous positive airway pressure (CPAP) respiratory support at the delivery room and subsequently were admitted to the neonatal intensive care unit. The vital parameters were monitored, with continued CPAP respiratory support. The treatment consisted of sedative, circulatory, and analgesic drugs, and total parenteral nutrition was initiated. The chest X-ray of the boy showed signs of pneumonia, while for the girl the X-ray was normal. There was no need to administer the surfactant. Additional tests showed elevated infection parameters; CRP 15,22 mg/L serum procalcitonin (PCT) 21,23 ng/mL for the boy in the 2nd day of life, while for the girl the levels were normal. The cultural swabs (pharynx, anal) taken at delivery for the boy showed *Enterococcus faecalis* HLAR, while the blood culture was negative. The cultural swabs from ear and pharynx for the girl were negative, while the blood culture showed methicillin-resistant staphylococcus (MRS). Since the infection parameters were normal and there were no clinical signs of sepsis, the bacteremia was diagnosed. The treatment consisted of a broad spectrum antibiotic therapy (ampicillin, amikacin) and immunoglobulins (Pentaglobin) after obtaining the blood culture results, the treatment of the girl was modified, with ampicillin replaced by vancomycin. In the following days of treatment, the children's condition was stable. The nasal CPAP respiratory support was continued for 6 days, with an additional day of oxygen. The routinely administered ultrasound head examinations showed no abnormalities. The neurological assessment of infants, apart from hypotonia of prematurity, showed the neuromotor development as normal for the age. Parenteral nutrition for both babies was continued for 9 days, and enteral feeding was started in the 3rd day of life, initially minimally, gradually increasing the dosage. The neonates were bottle fed with the maternal milk with human milk fortifier (HMF), and during the stay at the ward the breastfeeding was initiated. The babies were discharged at the age of 28 days (36 weeks corrected gestational age) with the weight of 2160 g for the baby girl and 2320 g for the baby boy. A follow-up examination 12 months later showed no major problems with apparent normal neurological development. The maternal postpartum course was uneventful; she stayed in hospital taking care of the babies.

## 3. Discussion

Since the first case of interval delayed delivery described by Carson in 1880, a number of studies concerning this subject have been reported [2–20]. Most of them were case reports; some presented maternal outcome and short-term outcome of newborns comparing time of delay, birth weight, gestational age, morbidity, and mortality (survival) in the first baby born prematurely and in the delayed sibling(s). Most of the reports demonstrated that delaying the delivery of the second twin or higher order multiples improves neonatal survival and gives higher birth weight. In a large population-based study, Zhang et al. reported that one week of delay in delivery was associated with an increase in infant birth weight of 131 g on average [3]. In the study of Farkouh et al., twenty-four patients had delayed interval deliveries resulting in a mean latency of 36 days with a range of 3 to 123 days and a significantly decreased perinatal mortality rate in the retained sibling group [5]. Most of the studies are focusing on pregnancy course and maternal outcome. The first study that assesses both the short- and long-term outcome in infants born after delayed interval delivery was the report of Rosbergen et al. in 2005 [6]. He compared 2 groups: group 1 consisting of first born neonates and group 2 consisting of the siblings, born after the delaying procedure. The “delayed infants” group was compared also to a reference group. The mean delay of delivery was 19,9 days (ranging from 2 to 75 days), which accounted for a significant increase in birth weight and neonatal survival. The number of diseases per infant (respiratory distress syndrome (RDS), bronchopulmonary disease (BPD), persistent ductus arteriosus (PDA), sepsis, peri- or intraventricular hemorrhage (PIVH), periventrivular leucomalacia (PVL), and retinopathy of prematurity (ROP)) decreased significantly; however, a significant difference between groups in the prevalence of the separate disease was not demonstrated. The authors did not show a negative effect on long-term development. The reference group showed fewer cases of sepsis than the delayed infants group [6].

To improve maternal and neonatal outcome in these pregnancies, methods of treatment in multiple pregnancies were discussed in a number of papers. There are no explicit guidelines as to how to practise in such cases; thus, treatment plans for such pregnancies are particularly challenging. To delay the delivery of subsequent fetus(es) in multifetal pregnancies, multiple therapies including prophylactic antibiotics, cervical cerclage, tocolysis, corticosteroids, and bed rest were used. The use of antibiotics was widely practised in the majority of the published cases. The use of tocolysis alone is not sufficient to prevent uterine contractions due to the premature rupture of the membranes and intrauterine infections, so most of the authors agree that antibiotics should also be used [4, 7]. In our case, the use of antibiotics in pregnancy improved the outcome of remaining fetuses. The usage of cervical cerclage is controversial because of the risk of chorioamnionitis and the premature rupture of the membranes [7]; this procedure may also stimulate uterus contractions. Most authors agree that if the membranes do not prolapse, an attempt to prolong gestation without cerclage should be considered [8, 9]. Others regard the cervical cerclage as helpful to prolong an interdelivery period. Petousis et al. demonstrated the effectiveness of emergency cervical cerclage in five cases of dichorionic twin pregnancies in order to achieve delayed interval delivery after the miscarriage of the first fetus (<24 weeks of gestation) [10]. It is recommended that women should stay in bed for the rest of their pregnancy; in our case, strict bed rest was also applied apart from other medical procedures (antibiotics, tocolysis, and corticosteroids). Irrespective of a good prenatal care this procedure may carry a risk for the pregnant mother; Roman et al. described 31.6% incidence of serious maternal morbidity related to the procedure [19]. In our case, the patient suffered no postpartum complications and awaited her children at maternity ward (similar to the study of Reinhart et al. in which no severe maternal complications had been observed [20]).

In the paper from 1998, Kalchbrenner presented the criteria and a protocol for delayed delivery. All patients who met the following criteria were considered to be candidates for active intervention: (1) multiple gestation with delivery of the first fetus occurring between 18 and 28 weeks' gestation; (2) diamniotic relationship between the initial and subsequent fetus or fetuses; (3) intact membranes in the remaining gestational sac or sacs; (4) absence of fetal distress, abruption of placentae, intra-amniotic infection, or maternal indication for delivery.

In our case, all criteria were fulfilled. On the basis of his experience of 7 multifetal pregnancies with delayed delivery, Kalchbrenner et al. demonstrated a clinically significant improvement in the outcome for the later-born neonates after an aggressive attempt to delay delivery. The interval between the initial and subsequent deliveries ranged from 2 to 92 days, with a mean of 32.6 days. The survival rate of firstborns (group 1) was 57%, but for the later-born infants (group 2), it stood at 78%. He also compared birth weight, gestational age (lower in group 1 than in group 2), the duration of respiratory support required for the neonates (longer in group 1) and the number of surgical procedures required during the hospitalized period (5 timed greater for group 1). The frequencies of necrotizing enterocolitis, patient ductus arteriosus, and retinopathy of prematurity were lower in group 2 than in group 1 [11]. The contraindications for the procedure include nonreassuring fetal status, congenital abnormalities, the rupture of the membranes of the remaining fetus, chorioamnionitis, severe hemorrhage (which suggests impending placental abruption), and maternal disease [12]. According to Arabin and van Eyck, monochorionicity is not a contraindication to the performance of delayed interval delivery because after the delivery of the first multiple and clamping of the cord, there is no remaining risk for twin-to-twin transfusion [13].

Each case of delayed delivery is a unique medical situation that must be met with the best possible solution dependent on possible risk, parents' wishes, and contraindications [13, 14]. Monitoring such pregnancies should include the early detection of chorioamnionitis, recurrent preterm contractions, signs of impending abruption, and coagulation disorders [12].

Based on a large number of studies, it is well established that prolonging pregnancy reduces the morbidity of premature birth and reduces the costs associated with neonatal intensive care [15]. As the delayed delivery pregnancies still seem to be a difficult task for the obstetricians, it is important to provide a continuum of intensive care for the fetus and the neonate at a tertiary center of perinatal care in order to improve their short- and long-term outcomes. Although the results from previous papers concerning interdelivery intervals are encouraging, it is important to prevent higher order multiple pregnancies to improve perinatal survival [8]. On the other hand, we still need more reports assessing both short- and long-term outcomes in infants born after delayed-interval delivery because by this procedure mortality of the retained children might be exchanged for longstanding morbidity (like cerebral palsy, learning disabilities, etc.) [4].

## References

[1] Wen SW, Demissie K, Yang Q, Walker MC (2004). Maternal morbidity and obstetric complications in triplet pregnancies and quadruplet and higher-order multiple pregnancies. *American Journal of Obstetrics & Gynecology*.

[2] Lipitz S, Reichman B, Paret G (1989). The improving outcome of triplet pregnancies. *American Journal of Obstetrics & Gynecology*.

[3] Zhang J, Hamilton B, Martin J, Trumble A (2004). Delayed interval delivery and infant survival: a population-based study. *American Journal of Obstetrics & Gynecology*.

[4] Yuce MA, Aybatli A, Kaplan PB (2010). Delayed-interval delivery of an in vitro-fertilized triplet pregnancy with premature rupture of membranes in the second trimester. *Archives of Gynecology and Obstetrics*.

[5] Farkouh LJ, Sabin ED, Heyborne KD, Lindsay LG, Porreco RP (2000). Delayed-interval delivery: extended series from a single maternal-fetal medicine practice. *American Journal of Obstetrics & Gynecology*.

[6] Rosbergen M, Vogt HP, Baerts W (2005). Long-term and short-term outcome after delayed-interval delivery in multi-fetal pregnancies. *European Journal of Obstetrics & Gynecology and Reproductive Biology*.

[7] Abboud P, Gallais A, Janky E (1997). Intentional delayed delivery in twin pregnancy two additional cases and literature review. *European Journal of Obstetrics & Gynecology and Reproductive Biology*.

[8] Olatunbosun OA, Turnell RW, Sankaran K, Ninan A (1995). Delayed interval delivery in quadruplets. *International Journal of Gynecology and Obstetrics*.

[9] Matalliotakis IM, Makrigiannakis AS, Giannakopoulou CC, Neonaki MA, Goumenou AG, Koumantakis EE (1998). Delayed interval delivery and survival of the two fetuses after second trimester loss of one triplet. *European Journal of Obstetrics & Gynecology and Reproductive Biology*.

[10] Petousis S, Goutzioulis A, Margioula-Siarkou C, Katsamagkas T, Kalogiannidis I, Agorastos T (2012). Emergency cervical cerclage after miscarriage of the first fetus in dichorionic twin pregnancies: obstetric and neonatal outcomes of delayed delivery interval. *Archives of Gynecology and Obstetrics*.

[11] Kalchbrenner MA, Weisenborn EJ, Chyu JK, Kaufman HK, Losure TA (1998). Delayed delivery of multiple gestations: maternal and neonatal outcomes. *American Journal of Obstetrics & Gynecology*.

[12] Kaneko M, Kawagoe Y, Oonishi J, Yamada N, Sameshima H, Ikenoue T (2012). Case report and review of delayed-interval delivery for dichorionic, diamniotic twins with normal development. *Journal of Obstetrics and Gynaecology Research*.

[13] Arabin B, van Eyck J (2009). Delayed-interval delivery in twin and triplet pregnancies: 17 years of experience in 1 perinatal center. *American Journal of Obstetrics & Gynecology*.

[14] Klearhou N, Mamopoulos A, Pepes S, Daniilidis A, Rousso D, Karagiannis V (2007). Delayed interval delivery in twin pregnancy: a case report. *Hippokratia*.

[15] Kao S-P, Hsu S, Ding D-C (2006). Delayed interval delivery in a triplet pregnancy. *Journal of the Chinese Medical Association*.

[16] Clerici G, Cutuli A, Renzo GCD (2001). Delayed interval delivery of a second twin. *European Journal of Obstetrics & Gynecology and Reproductive Biology*.

[17] Song T-B, Jeong J, Kim Y-H, Kim E-K (2000). Delayed interval delivery in multiple gestations. *Archives of Gynecology and Obstetrics*.

[18] Fayad S, Bongain A, Holhfeld P (2003). Delayed delivery of second twin: a multicentre study of 35 cases. *European Journal of Obstetrics & Gynecology and Reproductive Biology*.

[19] Roman AS, Fishman S, Fox N, Klauser C, Saltzman D, Rebarber A (2011). Maternal and neonatal outcomes after delayed-interval delivery of multifetal pregnancies. *American Journal of Perinatology*.

[20] Reinhard J, Reichenbach L, Ernst T (2012). Delayed interval delivery in twin and triplet pregnancies: 6 years of experience in one perinatal center. *Journal of Perinatal Medicine*.

